# Supplementary material for: Gramtools enables multiscale variation analysis with genome graphs
Source: Genome Biol. 2021 Sep 6;22:259. doi: 10.1186/s13059-021-02474-0 (PMC8420074; doi:10.1186/s13059-021-02474-0)
Supplement: Supplementary file 1 — Additional file 1 Contains all supplementary text and figures for this paper. [file 13059_2021_2474_MOESM1_ESM.pdf]

Supplementary material for *Enabling multiscale variation analysis with genome graphs*

Brice Letcher, Martin Hunt and Zamin Iqbal

August 10, 2021

Contents

Multiscale variation 2
Graph constraints 3
Validation of nested genotyping with simulated data 8
Benchmarking `gramtools` genotyping against single-reference variant callers at surface antigens 9
Unified SNP and large deletion analysis in *M. tuberculosis* 15
Application: charting SNPs on top of alternate haplotypes 20
JSON VCF (jVCF) format specification 23

Multiscale variation

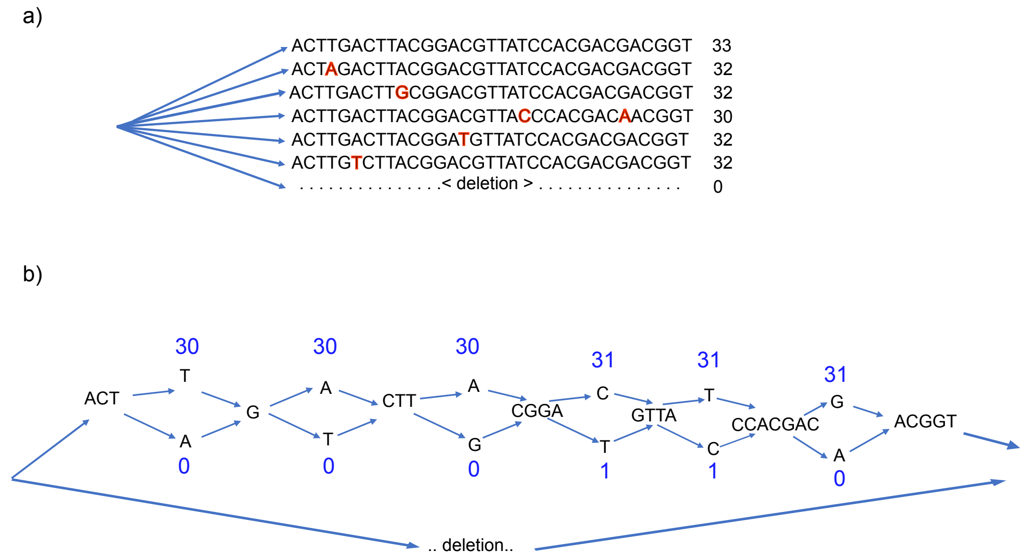

Fig. S1: **Comparison of genotyping in non-nested and nested DAGs.** This is intended to show the impact of nesting on genotyping without representing any specific implementation. a) Non-nested graph showing a site with several long alternate alleles differing by SNPs (shown in red). Top allele is the actual allele in a sequenced genome, whose reads have been mapped to this graph. On the right hand end of each allele is the average depth of coverage on this allele. The bottom allele is a deletion, shown with zero coverage. There is very little difference between the true allele and the others which differ by SNPs but only have marginally lower depth of coverage when averaged across the allele. Confidence of the called allele will be low. b) The same sequences in a nested graph, with coverage shown at nested sites. Precise coverage at SNPs makes it very clear what is going on - all SNP calls will be high confidence and correct.

In Fig. S1, we refer to nested and non-nested DAGs; these are formally defined in the Methods section of the paper.

Graph constraints

In this section, we illustrate the types of graphs supported by `gramtools`. In Fig. S2, we illustrate how variant sites are mapped on DAGs: from an opening node v (shown in blue), the nodes on all paths from v to the graph's sink node form a set S that is non-empty and totally ordered. The minimal element of S is the closing node $c(v)$ of v (see Methods in the main section for details).

In Fig. S3, we illustrate the *balanced bracket* property and graphs with and without this property. Graphs without a balanced brackets representation cannot be indexed by `gramtools` (see Methods in the main section). As shown in Fig. S3, graphs without this property can sometimes be transformed into equivalent graphs (ie representing the same sequence) with this property.

In Fig. S4, we show a graph with a balanced brackets representation that cannot be directly indexed by `gramtools`. `gramtools` requires a one-to-one correspondence between the opening and closing nodes of the graph (ie each opening or closing node cannot open or close more than one variant site). In Fig. S4 however, node 1 is the opening node for both Site 1 and Site 2. To make the graph indexable by `gramtools`, each bracket node in the balanced bracket representation of the graph is added to the graph, yielding the graph at the bottom. This graph has a one-to-one correspondence between opening and closing nodes and can thus be indexed by `gramtools`.

The Methods part of the main section formally describes how to produce a balanced brackets representation and how to transform a graph with balanced brackets into a `gramtools`-indexable graph.

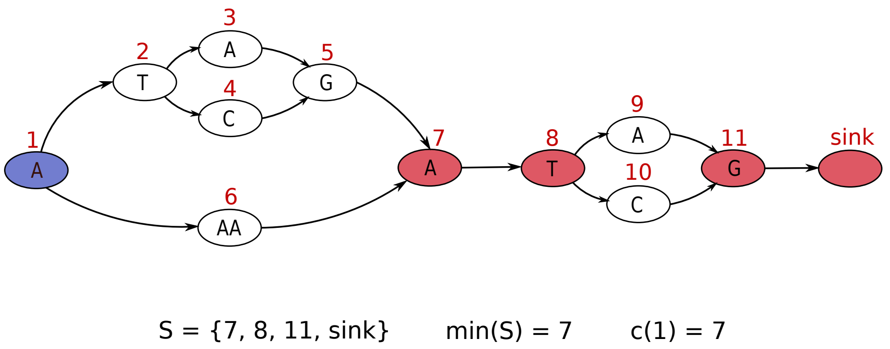

Fig. S2: **Illustration of variant site definition in a DAG.** Given an opening node (node 1, shown in blue), we call S the set of nodes on all paths from v to the sink (shown in red). This set is non-empty and totally ordered and so has a minimal element, which is node 7 in this example. Therefore node 7 is the closing node of node 1, and the subgraph induced from node 1 to 7 forms a variant site.

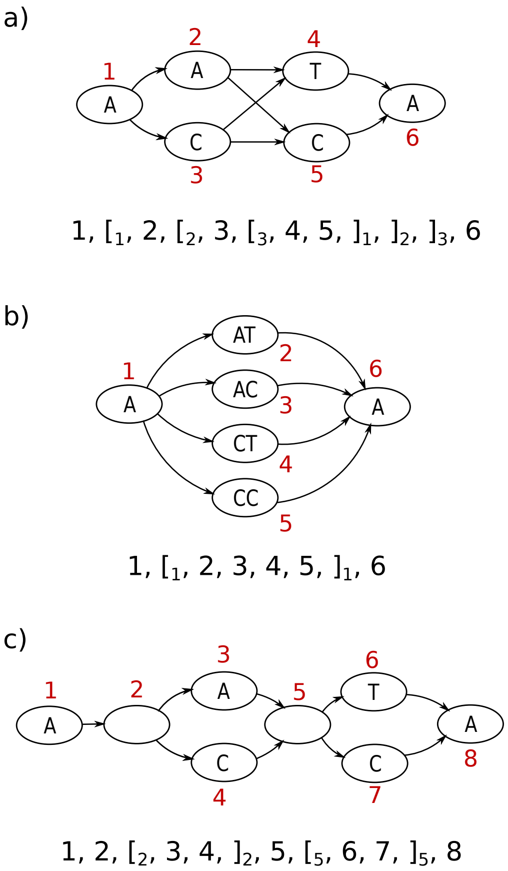

Fig. S3: **Indexable and non-indexable graphs representing equivalent sequences.** The graphs in the three panels represent the same set of sequences but have different topology. Each node is labeled with an integer identifier in red, increasing according to a topological ordering of the nodes. The balanced brackets representation of each graph is written below it. The graph in panel a) does not have balanced brackets and cannot be indexed by `gramtools`, while the graphs in panels b) and c) do and can therefore be indexed.

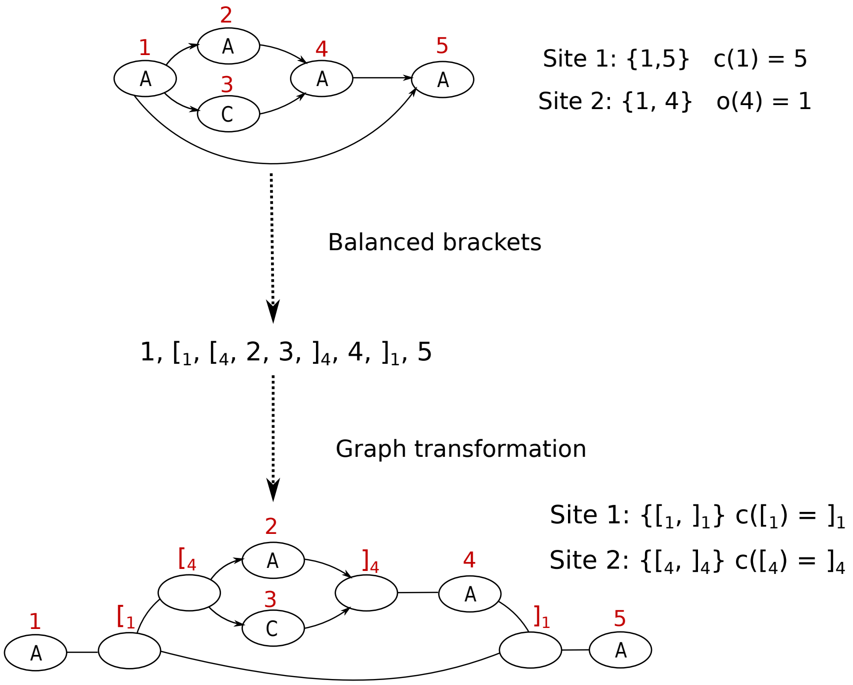

Fig. S4: **`gramtools` transformation of indexable graphs.** Each node is labeled with an integer identifier in red, increasing according to a topological ordering of the nodes. The graphs each have two variant sites, with opening and closing nodes marked between curly brackets. In the top graph, node 1 is the opening node for both site 1 ($c(1) = 5$ is the closing node of site 1) and site 2 ($o(4) = 1$ is the opening node of site 2). Thus `gramtools` cannot index this graph, as a one-to-one correspondence between opening and closing nodes is required. From the balanced bracket representation of this graph, a new `gramtools`-indexable graph is produced, by adding one node for each bracket to the graph (see Methods in the main section for how). In this new graph, each site has a distinct pair of opening and closing nodes.

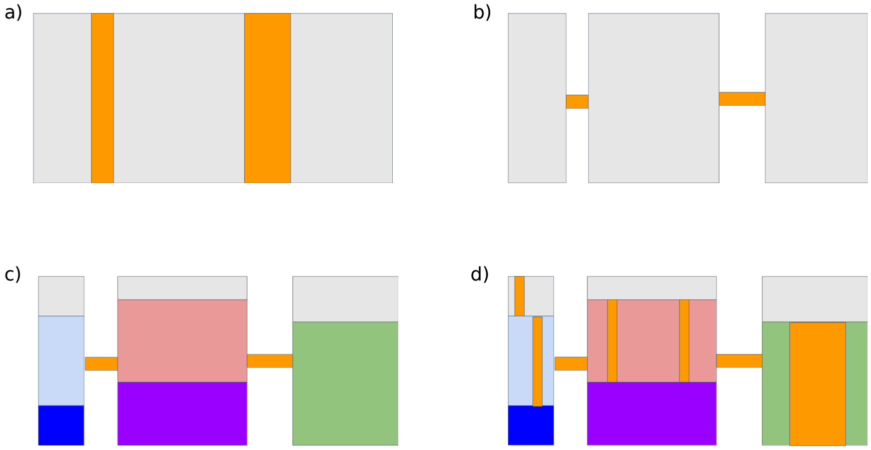

Fig. S5: **Illustration of the recursive collapse and cluster (RCC) algorithm for graph construction.** The input to the algorithm is a multiple-sequence alignment (MSA), shown as the spanning grey rectangle in all panels. **a)** Collapse operation. Regions of MSA of at least a given size where all samples have the same sequence are identified, shown as orange strips. **b)** These become invariant sequence nodes in the output graph. Each of the three resulting grey blocks represents one variant site and will be treated independently. **c)** Cluster operation. For each variant site from a/b, sequences are clustered using k-means, shown as one coloured block per cluster. **d)** Recursive application of RCC to each coloured block (here, only the collapse operation is shown). When the length of the coloured block becomes too short, or no suitable clustering is found, all sequences are simply enumerated as alternate alleles, ending the recursion.

In this paper, gramtools takes as input graphs that are NDAGs by construction. These are built using our tool make_prg from multiple-sequence alignments (MSA). In Fig. S5, we illustrate the algorithm implemented in make_prg. Notice that in this figure, each coloured block corresponds to one variant site, with blocks occurring inside other blocks. If we represent each block using a pair of matching opening and closing brackets, enumerating the brackets by traversing the MSA from left to right and top to bottom, it is clear that the entire set of brackets is balanced. Further, make_prg builds graphs with a one-to-one correspondence between opening and closing nodes of variant sites. Thus, the graphs it constructs are gramtools-indexable NDAGs.

Validation of nested genotyping with simulated data

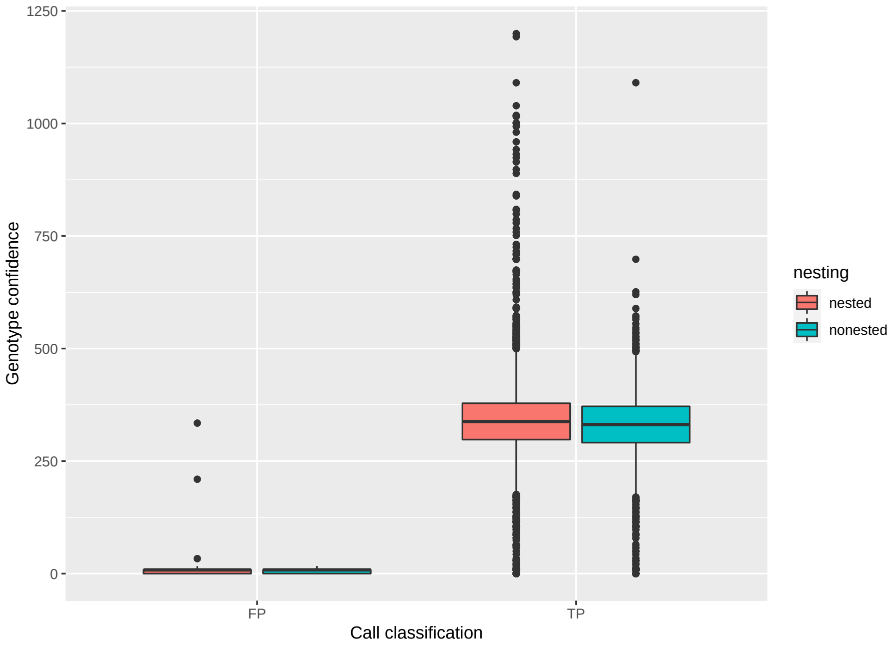

Fig. S6: **Improved genotype confidence on nested graphs.** The distribution of genotype confidence (GC) is shown for true positive (TP) and false positive (FP) calls. An FP call counts when the wrong call is made or no call should have been made. Lower overall confidence of FPs allows for filtering.

Benchmarking `gramtools` genotyping against single-reference variant callers at surface antigens

We provide here a breakdown of performance for each gene, which also includes the performance of running `SAMtools` and `Cortex` on top of the `gramtools`-inferred personalised reference (PR) genome for each sample (which is the path given by the maximum-likelihood allele at each variant site). In AMA1, the gene is easily resolved and performance is the same for all three tools (Fig. S7), while in EBA175, DBLMSP and DBLMSP2, `gramtools` outperforms the single-reference callers (Fig. S8, 9, 10). Running `SAMtools` and `Cortex` against the gramtools-inferred PR unlocks further variation in one gene, DBLMSP2 (Fig. S10), validating the use of a PR for discovering new variation. In DBLMSP, four samples remain at large distances of (4-10%) to the truth assemblies for all tools (Fig. S9). These sequences could also not be resolved by `SAMtools` and `Cortex`, suggesting long-read sequencing would be required to fully access the variation in these four samples.

Comparing `gramtools` genotyping with taking the best input sequence in the graph

One advantage of using graphs for genotyping is that the inferred sample's path through the graph can be a recombinant of the sequences used to build the graph. Using our 14 validation samples, we measured to what extent `gramtools` can recover the closest possible sequence out of the input sequences used to construct the graph, and how often it found a recombinant that is better than this closest sequence (note that all input sequences exist as paths in the graph). The closest sequence for a gene in a sample was determined by mapping all 2,498 sequences used for building the gene's graph to the sample's truth assembly with `bowtie2`. The closest sequence was the one with the lowest edit distance (NM tag), provided it had mapping quality (MAPQ) greater than 20 (i.e. the probability the mapping was not unique was $< 10^{-2}$ according to `bowtie2`).

We show the results in Fig.S11, plotting the distance of the best panel reference from the truth, and the distance of the `gramtools`-inferred sequence from the truth (where truth refers to the sequence in the truth assembly of the relevant sample). Sequences below the x=y dotted line indicate recombinants found by `gramtools` that are closer to the truth assembly than any input to the graph. In AMA1 and EBA175, the `gramtools` inference was as good or closer to the truth for 13/14 samples. For DBLMSP2, `gramtools` finds many (8) recombinants in the graph not present in the input sequences. For DBLMSP, in two samples the `gramtools` inference is much worse than the best input in the graph, indicating genotyping failure. We found this was due to a specific unresolved region where sites had null calls caused by low coverage.

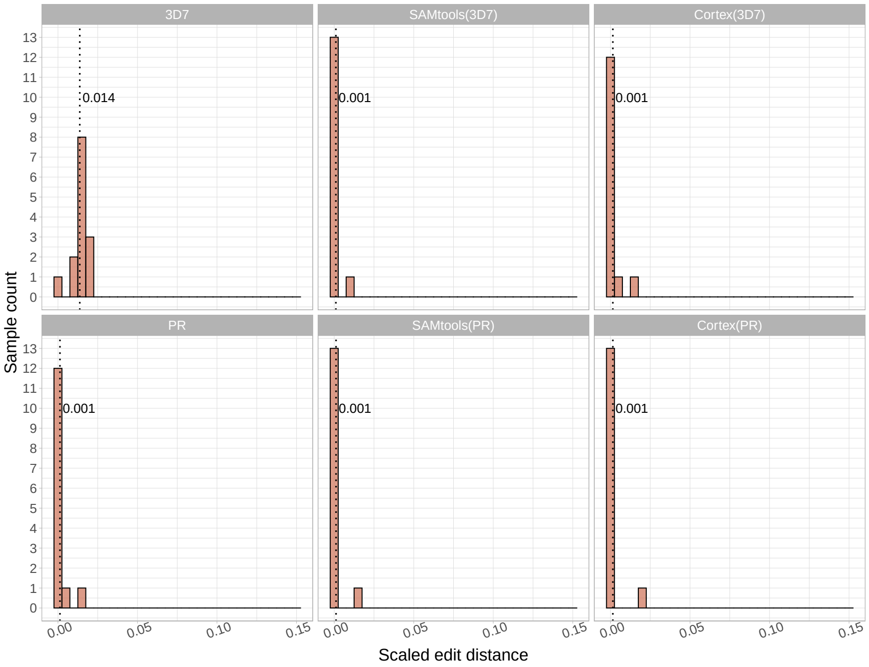

Fig. S7: **`gramtools` genotyping compared to reference-based variant calling in AMA1.**The x-axis is the scaled edit distance (edit distance divided by the length of the gene) to the true sequence as determined from high-quality assemblies, and the y-axis shows counts for the 14 evaluated samples. The two histograms in the left-hand column show how close the 3D7 reference genome and the `gramtools`-inferred personalised reference (PR) are to each sample. The two histograms in the middle column show results for `SAMtools` when using the 3D7 and PR as reference genome. The histograms in the right-hand column are the equivalent plots for `Cortex`. The dotted lines and adjacent numbers show the mean scaled edit distance.

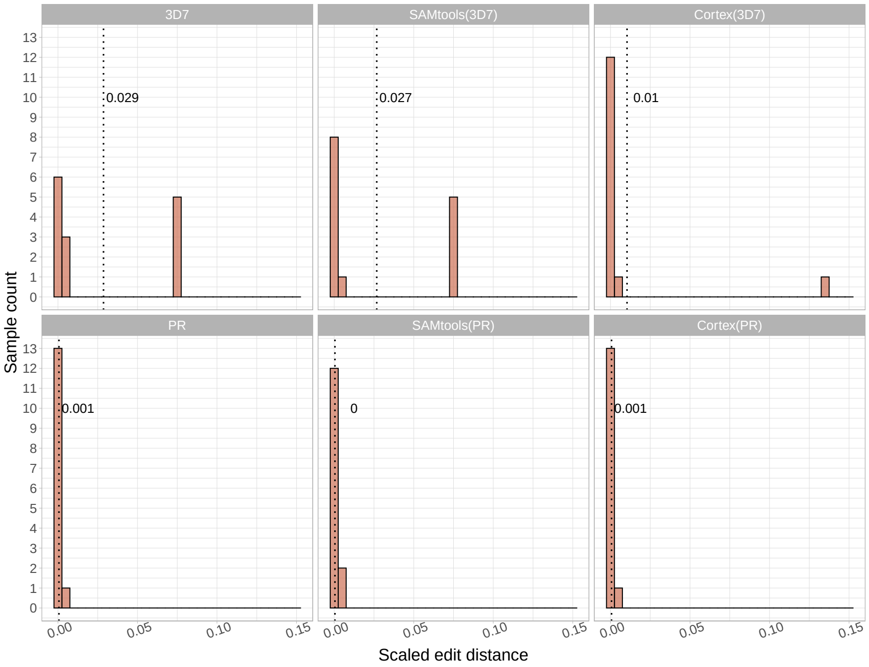

Fig. S8: **`gramtools` genotyping compared to reference-based variant calling in EBA175.** The x-axis is the scaled edit distance (edit distance divided by the length of the gene) to the true sequence as determined from high-quality assemblies, and the y-axis shows counts for the 14 evaluated samples. The two histograms in the left-hand column show how close the 3D7 reference genome and the `gramtools`-inferred personalised reference (PR) are to each sample. The two histograms in the middle column show results for `SAMtools` when using the 3D7 and PR as reference genome. The histograms in the right-hand column are the equivalent plots for `Cortex`. The dotted lines and adjacent numbers show the mean scaled edit distance.

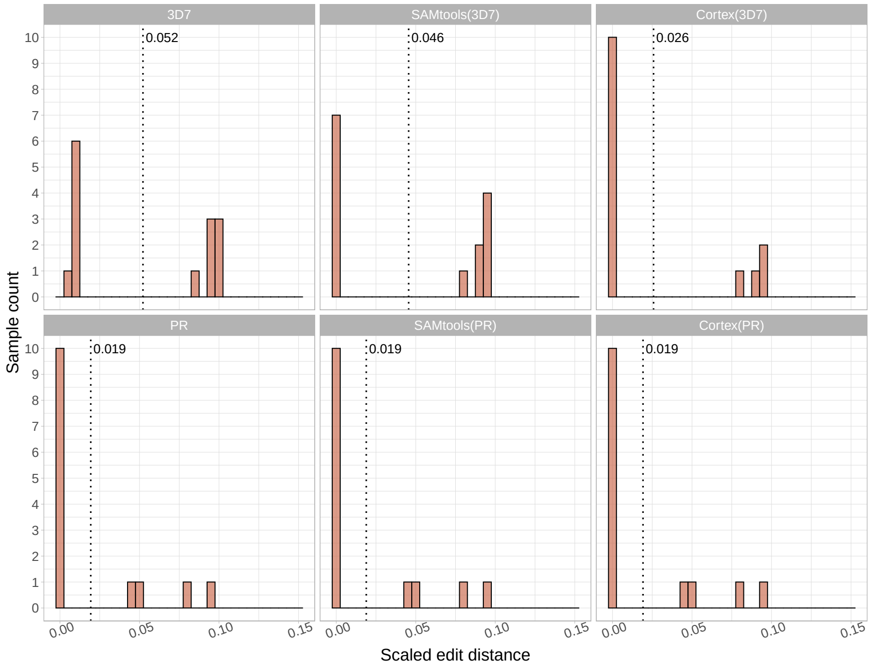

Fig. S9: **`gramtools` genotyping compared to reference-based variant calling in DBLMSP.** The x-axis is the scaled edit distance (edit distance divided by the length of the gene) to the true sequence as determined from high-quality assemblies, and the y-axis shows counts for the 14 evaluated samples. The two histograms in the left-hand column show how close the 3D7 reference genome and the `gramtools`-inferred personalised reference (PR) are to each sample. The two histograms in the middle column show results for `SAMtools` when using the 3D7 and PR as reference genome. The histograms in the right-hand column are the equivalent plots for `Cortex`. The dotted lines and adjacent numbers show the mean scaled edit distance.

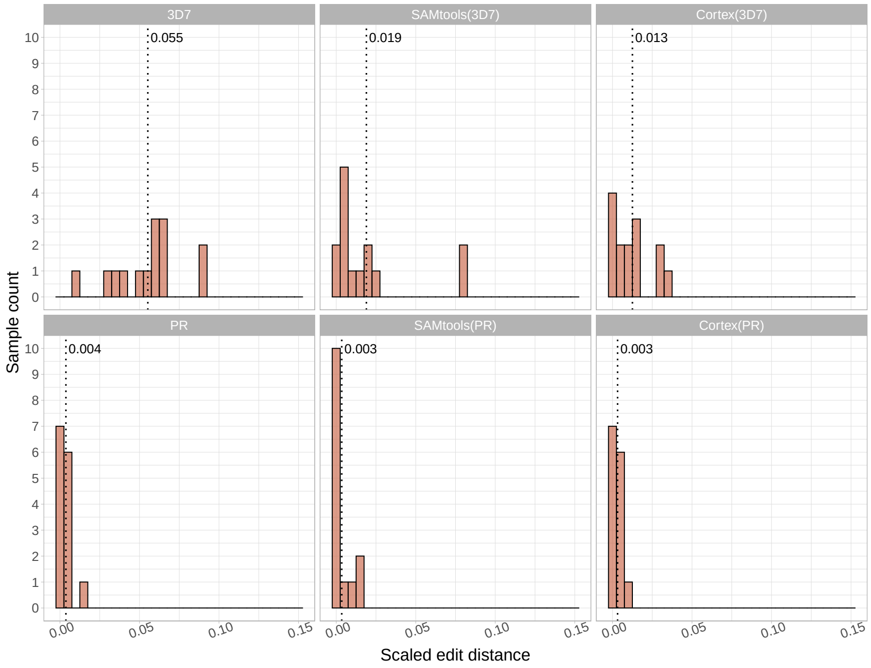

Fig. S10: **`gramtools` genotyping compared to reference-based variant calling in DBLMSP2.** The x-axis is the scaled edit distance (edit distance divided by the length of the gene) to the true sequence as determined from high-quality assemblies, and the y-axis shows counts for the 14 evaluated samples. The two histograms in the left-hand column show how close the 3D7 reference genome and the `gramtools`-inferred personalised reference (PR) are to each sample. The two histograms in the middle column show results for `SAMtools` when using the 3D7 and PR as reference genome. The histograms in the right-hand column are the equivalent plots for `Cortex`. The dotted lines and adjacent numbers show the mean scaled edit distance.

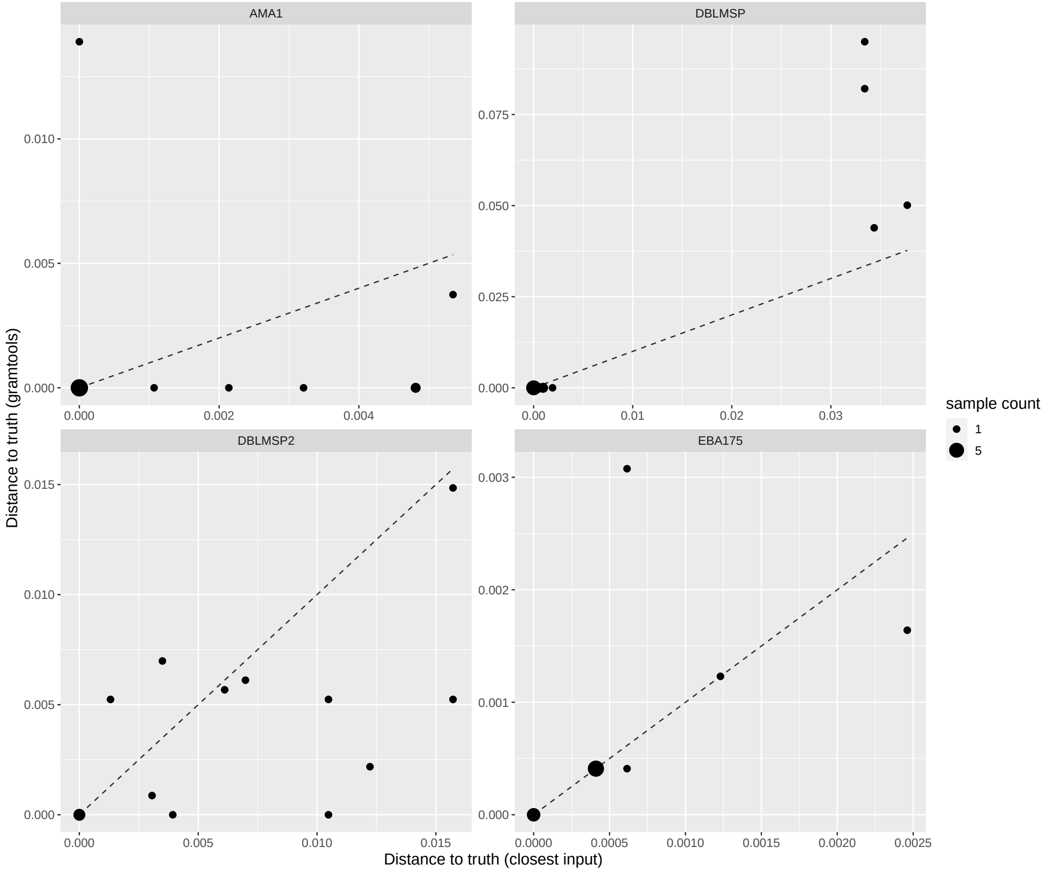

Fig. S11: `gramtools` **genotyping finds recombinants between input haplotypes in the graph.** Each dot shows one gene in one sample, with each gene in its own panel. The x-axis is the edit distance between the closest input sequence (out of all sequences used to construct the `gramtools` genome graph) and the truth assembly, divided by the length of the gene. The y-axis shows the edit distance between the `gramtools`-inferred sequence and the truth assembly. The dotted y=x line shows the expectation if `gramtools` always found in the graph the closest sequence used in graph construction. Points above this line highlight `gramtools` genotyping errors, as the closest input should have been found. Points below the line show `gramtools` finding recombinants in the graph that are closer than any input sequence used in graph construction.

Unified SNP and large deletion analysis in *M. tuberculosis*

Variant discovery

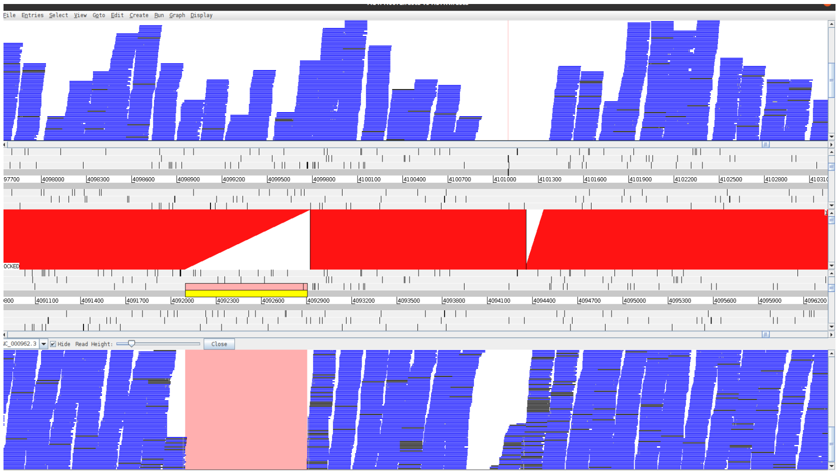

Fig. S12: **Manual confirmation of a deletion in sample N0072 using `ACT`.** The top track shows the assembly and the bottom track the reference. Mapped reads are shown as blue rectangles. Bright red rectangles show mapped regions between assembly and reference. The light red rectangle highlights the location of the confirmed deletion.

Mapping evaluated regions to truth assemblies

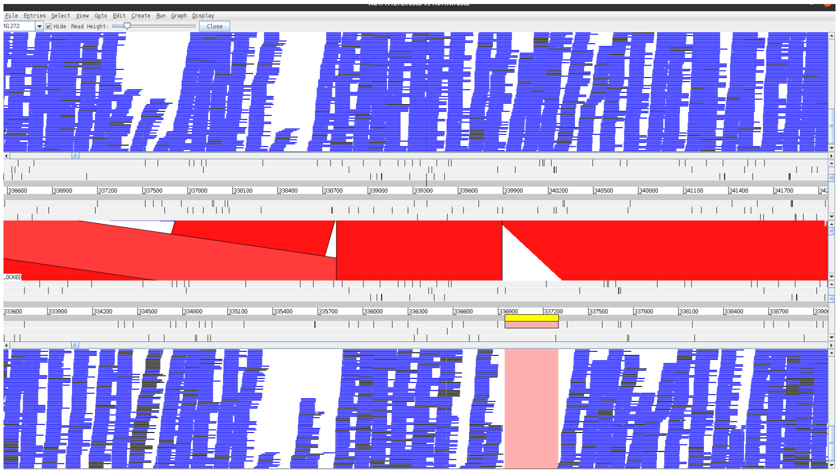

Fig. S13: **Manual confirmation of a deletion in sample N1272 using** `ACT`**.** The top track shows the assembly and the bottom track the reference. Mapped reads are shown as blue rectangles. Bright red rectangles show mapped regions between assembly and reference. The light red rectangle highlights the location of the confirmed deletion.

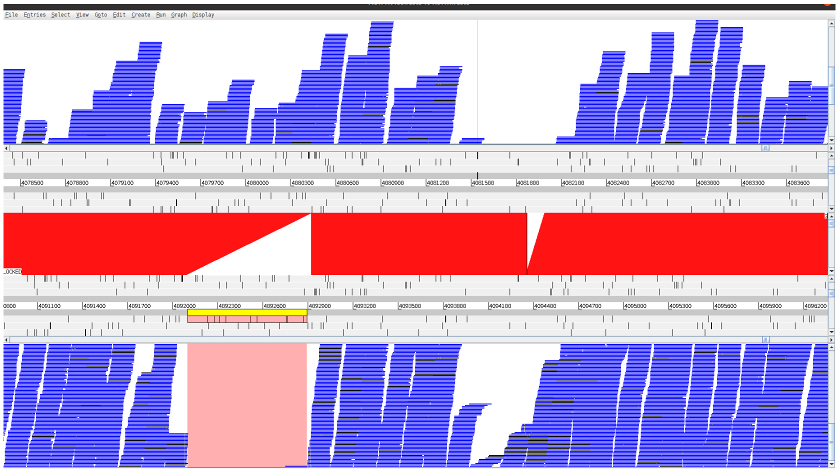

Fig. S14: **Manual confirmation of a deletion in sample N0153 using** `ACT`**.** The top track shows the assembly and the bottom track the reference. Mapped reads are shown as blue rectangles. Bright red rectangles show mapped regions between assembly and reference. The light red rectangle highlights the location of the confirmed deletion.

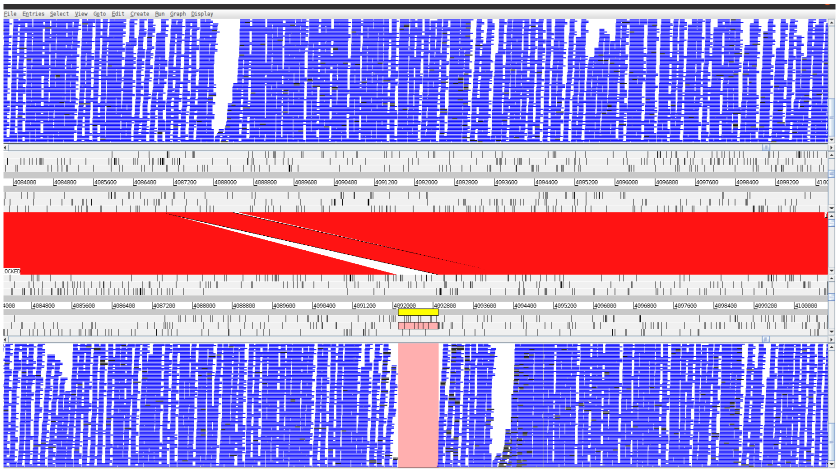

Fig. S15: **Manual confirmation of a deletion in sample N0157 using ACT.** The top track shows the assembly and the bottom track the reference. Mapped reads are shown as blue rectangles. Bright red rectangles show mapped regions between assembly and reference. The light red rectangle highlights the location of the confirmed deletion.

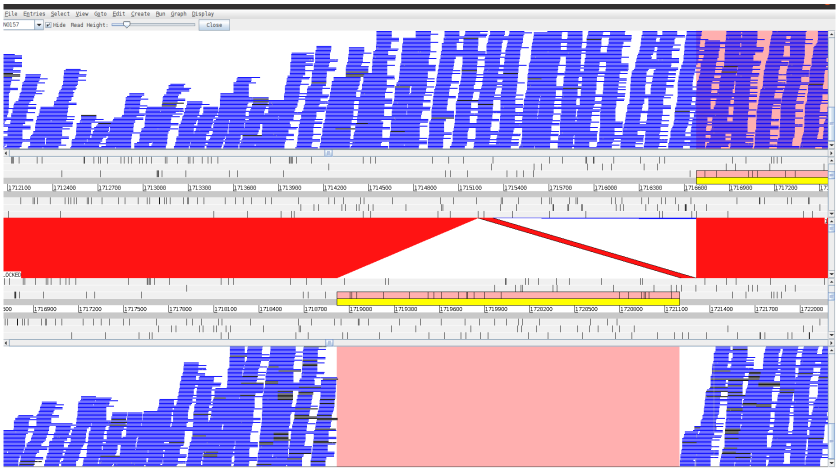

Fig. S16: **Manual confirmation of another deletion in sample N0157 using ACT.** The top track shows the assembly and the bottom track the reference. Mapped reads are shown as blue rectangles. Bright red rectangles show mapped regions between assembly and reference. The light red rectangle highlights the location of the confirmed deletion.

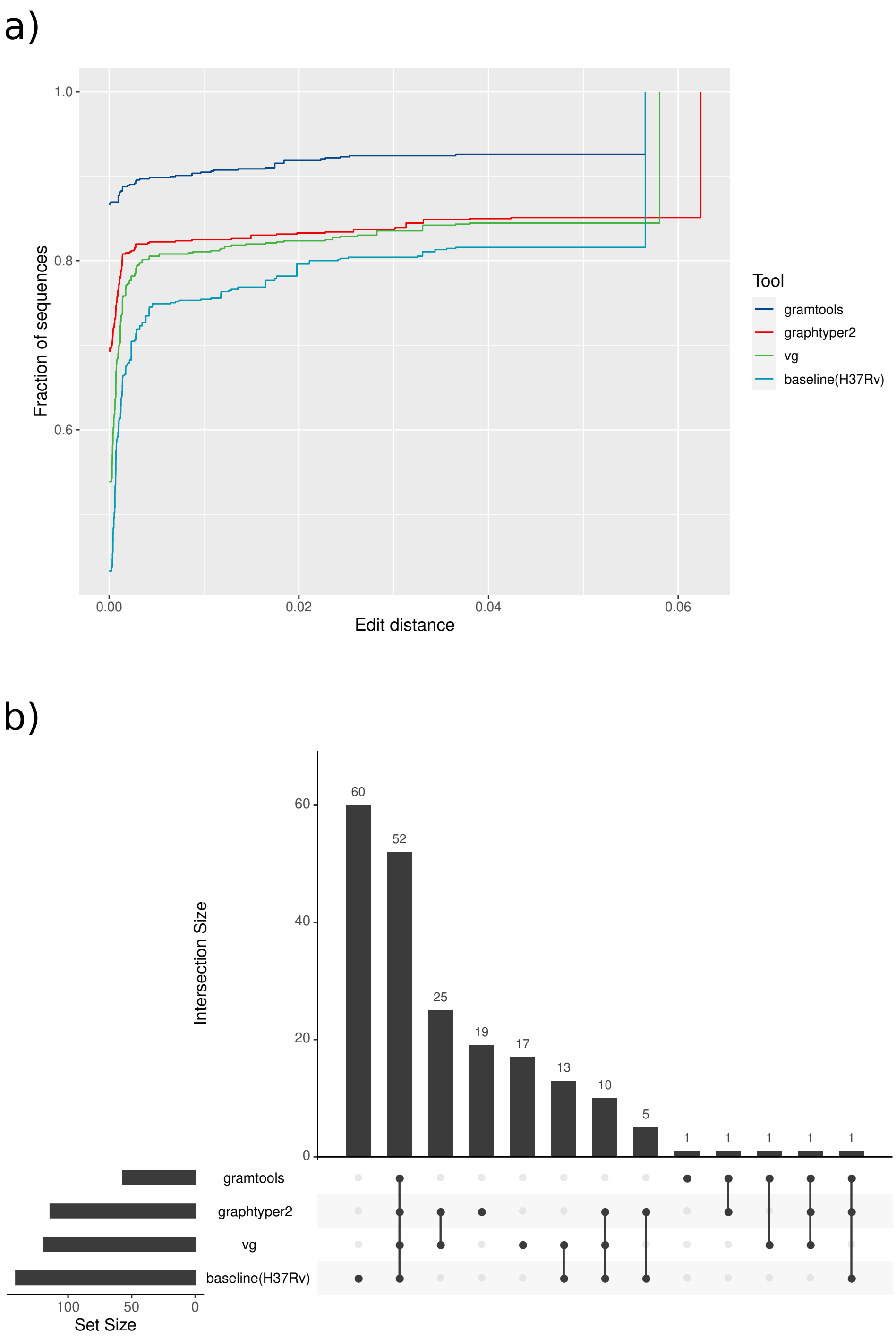

Fig. S17: **Joint SNP and deletion genotyping results using `bowtie2` alignments.** a) Cumulative distributions of edit distances between sequences and the truth assemblies. `bowtie2` can only align sequences up to 4.5% divergence making the fraction of aligned sequences lower. Mean edit distance is lowest for `gramtools`, followed by `GraphTyper2` and `vg`. b) Upset plot of unmapped and MAPQ $\leq$ 30 sequences. For a large number of sequences, the reference genome sequence does not align to the assembly illustrating high divergence between the two.

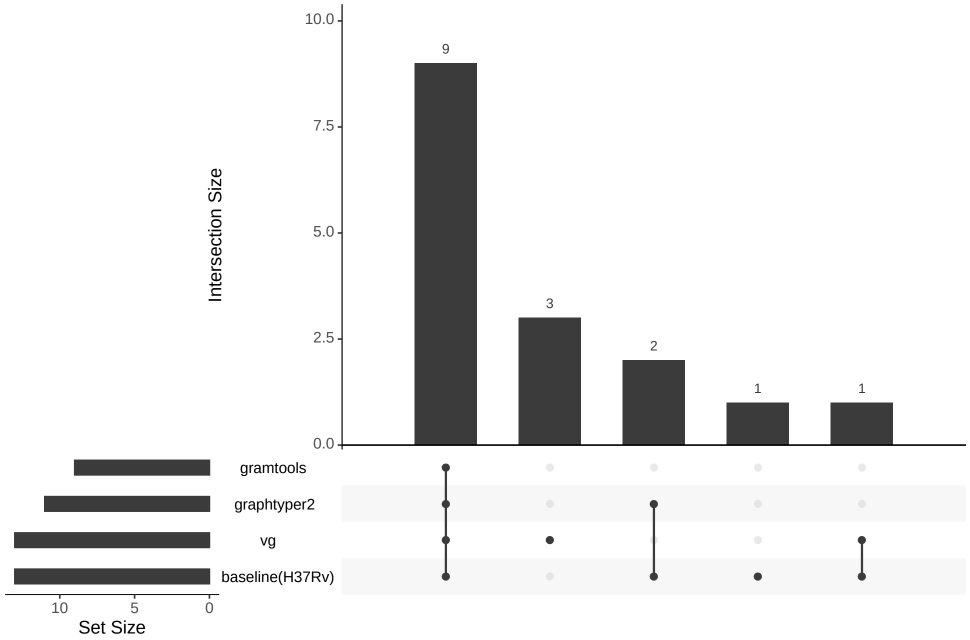

Fig. S18: **Unmapped and filtered out sequences in joint SNP and deletion genotyping experiment using `minimap2` based alignments.** Upset plot showing the total number of unmapped sequences and sequences with MAPQ ≤ 30 and their intersections by condition.

Evaluating variant calls using `varifier`

In the main section of the paper, we benchmark genotyping accuracy of genome graph tools for all variants combined inside a genomic region. Here, we show the genotyping accuracy of individual variants broken down by type (insertion, deletion, SNP) and size (1-10bp, 11-50bp, 51bp and above). To achieve this, we computed the precision and recall of individual variant calls using our tool `varifier` (see Methods in the main section).

Fig. S19 shows the precision and recall for each variant category. Each tool is genotyping a fixed set of input variants incorporated into a genome graph, without an additional discovery step. Thus recall in Fig. S19 is measured relative to the variants originally present in the input VCF files and validated as true (as any variants absent from the genotyped graphs cannot be expected to be called by the tools). The performance differences between the tools are discussed in the main section of the paper.

Application: charting SNPs on top of alternate haplotypes

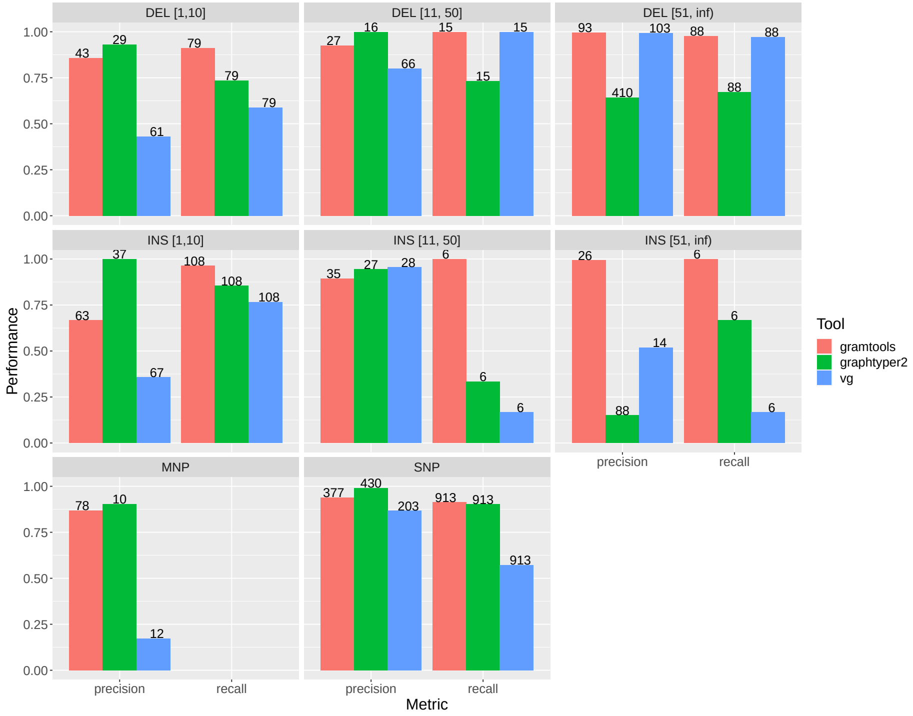

Fig. S19: **Breakdown of genotyping performance by type and size of variant for variants known to be in the graph.** For each variant category, genotyping precision (the fraction of calls made that were correct) and recall (the fraction of calls made that were expected) are shown (see Methods in the main section for how these are computed). Numbers above each bar show the number of evaluated variants. Recall is measured only on the set of variants validated as true in the input VCFs, and thus expected to be called by each tool. Precision is measured for all the calls made by each tool and not dependent on the validated input variants. INS: insertion, DEL: deletion, SNP: single-nucleotide polymorphism, MNP: multi-nucleotide polymorphism.[51, inf) means variants of size 51 bp up to an unlimited size.

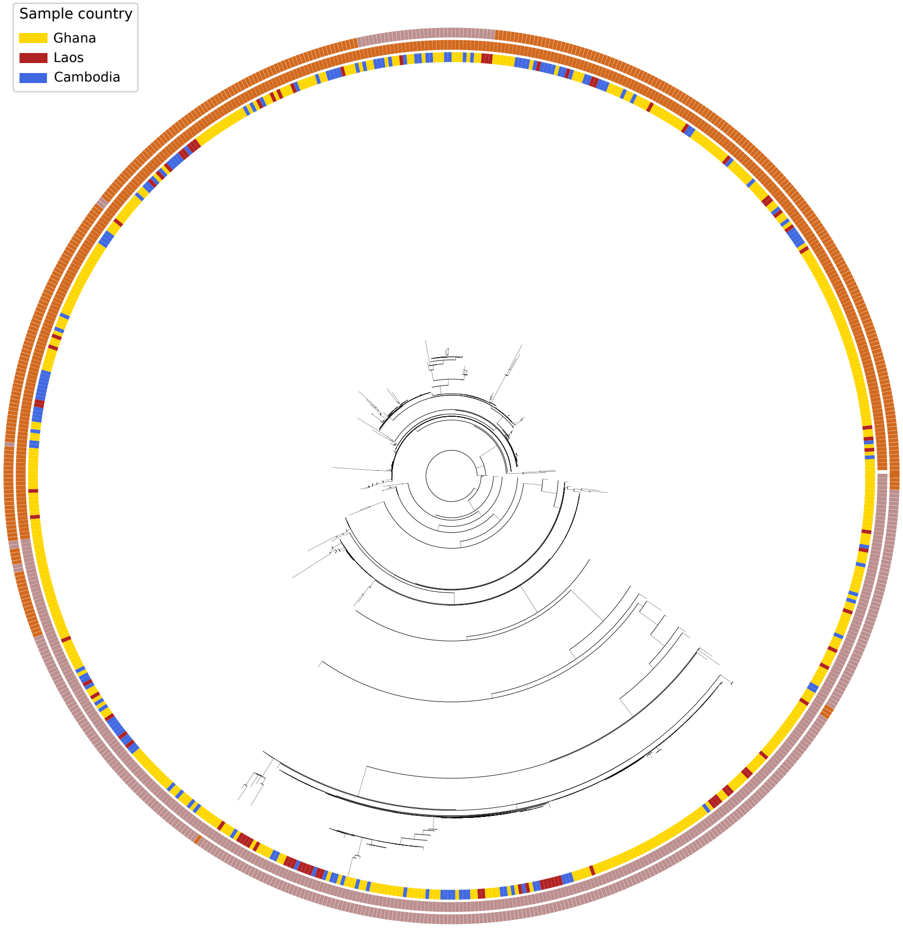

Fig. S20: **Phylogenetic tree of the DBL domain of DBLMSP2.** The inner circle colours samples by country of origin. The middle circle shows phylogeny-based assignment of samples into the two gene forms. The outer circle shows hierarchical clustering-based assignment of samples into the two forms, as used in the clustering tree of the figure in the main section. The assignments for the two approaches are largely the same.

JSON VCF (jVCF) format specification

The specification of the JSON Variant Call Format (jVCF) produced by `gramtools` is publicly available on the following repository: https://github.com/iqbal-lab-org/jVCF-spec.

We provide below the version corresponding to commit 28a955933baadd6c0536856433634c1d14f37ea8 of the repository.

JSON Variant Call Format Specification

Version 0.1

January 13, 2021

Contents

1 Rationale **2**

1.1 Variant calls in genome graphs 2

1.2 Requirements 2

1.3 Description of JSON 2

2 Example **3**

3 Specification **6**

3.1 Site_Fields 6

3.2 Sites 6

3.3 Samples 7

3.4 Filters 7

3.5 Model 8

3.6 Child_Map 8

3.7 Lvl1_Sites 8

1 Rationale

1.1 Variant calls in genome graphs

The Variant Call Format (VCF) is a tab-delimited data format describing genetic variation occurring with respect to a linear reference genome. Here we define an extension to VCF for genome graphs, which are graph structures representing genetic variants occurring on potentially multiple reference genomes.

The format uses JavaScript Object Notation (JSON), a commonly used data format, and we thus call it JSON VCF or jVCF.

1.2 Requirements

jVCF assumes:

- Variant sites have been defined on the genome graph
- Variant sites can be contained in other variant sites. It is known which sites are contained in which others.

For the latter point, a site contained in another occurs in a given sequence background. Each sequence background must be labeled with a unique positive integer, called its **haplogroup**. See this toy graph for an example.

1.3 Description of JSON

The JSON format is defined here: https://www.json.org/json-en.html.

Briefly, JSON consists of *objects* and *arrays*:

- An object is an unordered collection of key/value pairs enclosed in curly brackets ('{' and '}').
- An array is an ordered collection of values enclosed in square brackets ('[' and ']').

In an object, a key is a string. Strings are enclosed in double quotes ('"'). In objects and arrays, values can be:

- a string
- a number
- one of the literals 'true', 'false' or 'null'
- an array
- an object

2 Example

Here is a toy genome graph:

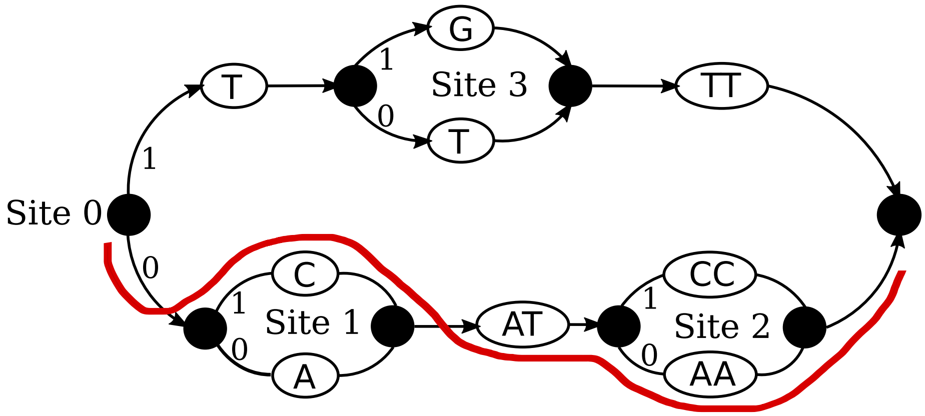

Figure 1: Genome graph with haploid genotyped sample as red path

In Figure 1, black nodes mark site entry and exit points. Each variant site is labeled by a unique positive integer ID, and each outgoing branch from the start of a variant site is labeled by a unique positive integer (its haplogroup).

Assume the ploidy is 1, and we are genotyping a sample whose genotyped path in the graph is the red line. The jVCF of this genotyped sample would look like this:

```
{
  "Sites":[
    {"ALS":["AATAA","CATAA"],"FT":[[]],"GT":[[1]],"HAPG":[[0]],"POS":1,"SEG":"myRef"},
    {"ALS":["A","C"],"FT":[[]],"GT":[[1]],"HAPG":[[1]],"POS":1,"SEG":"myRef"},
    {"ALS":["AA"],"FT":[[]],"GT":[[0]],"HAPG":[[0]],"POS":4,"SEG":"myRef"},
    {"ALS":["T"],"FT":[[]],"GT":[[null]],"HAPG":[[]],"POS":2,"SEG":"myRef"}
  ],
  "Site_Fields": {
    "ALS": {
      "Desc": "Alleles at this site"
    },
    "FT": {
      "Desc": "Filters failed in a sample"
    },
    "GT": {
      "Desc": "Genotype"
    },
    "HAPG": {
      "Desc": "Sample haplogroups of genotyped alleles"
    },
    "POS": {
      "Desc": "Position on reference or pseudo-reference"
    },
    "SEG": {
      "Desc": "Segment ID"
    }
  },
  "Samples": [
    {
      "Desc": "mySample description",
      "Name": "mySample"
    }
  ],
  "Filters": {
    "MINQ": {
      "Desc": "Call is below minimum quality"
    }
  },
  "Model": "myGenotypingModel",
  "Child_Map": {
    "0": {
      "0": [1,2],
      "1": [3]
    }
```

```
  },
  "Lvl1_Sites": [0]
}
```

3 Specification

There are 7 required keys in jVCF, which can appear in any order. You can use extra keys beyond the required ones. We list each of the required keys below, giving for each the type and description of its corresponding value.

3.1 Site_Fields

Type Object

Description Each key describes the fields that can appear in Site objects.

The following keys are required to be present:

Key	Meaning
ALS	Genotyped alleles at this site
SEG	Segment (eg chromosome) on which the site lies
POS	1-based position relative to haplogroup reference
GT	Genotype calls
HAPG	Haplogroup calls: haplogroup the called allele(s) lie on
FT	Filters set

Each key corresponds to an object with at least a “Desc” key which gives a description of the field. Extra keys beyond the required ones can be used.

3.2 Sites

Type Array

Description Each element is a Site object

3.2.1 Site objects

A Site object describes variant calls at one site. This is analogous to a record in VCF.

As for Site_Fields, the following keys are required to be present:

Key	Value Type
ALS	Array of strings
SEG	String
POS	Number
GT	Array of array of numbers, ‘null’ or empty
HAPG	Array of array of numbers or empty
FT	Array of arrays of strings or empty

Here is a more detailed explanation of the keys:

- `ALS`: does not need to store all possible alleles at the variant site. It must store at least one allele which is the reference for this site. The reference allele is the allele obtained by following haplogroup 0 from the start of the site to the end of the site. All called alleles should also be present in `ALS`.
- `SEG`: the name of the genomic segment the site lies on, e.g. chromosome or the name of a reference genome.
- `POS`: 1-based position, offset from the last non-nested site (see Lvl1_Sites). The position of sites contained in other sites is expressed relative to the reference path through the containing site, which is obtained by following haplogroup 0.
- `GT` and `HAPG`: are arrays of arrays. The two array levels are:

  1. Samples. This should have the same size as the array in Samples.
  2. Ploidy. This should have one entry per chromosome copy, or chromosome population (eg tumour subclones or mixed infections).

  For example, to access the first sample's genotype calls at a site, you access the 0th element of the `GT` array. If you have a diploid genotyped sample, the first and second chromosome calls are the 0th and 1st elements of that array.
- `FT`: also an array of arrays. The first level is the samples, and the second the filters set for that sample.

You can use more keys. For each used key, Site_Fields needs to provide a description of its meaning.

3.3 Samples

Type Array

Description Each entry is an object describing one genotyped sample.

Each entry must contain the following keys: "Name" and "Desc", giving the sample name and its description. The order of the samples matters: it must match that in Site objects.

3.4 Filters

Type Object

Description Describes the filters that can appear in Site objects.

Each filter that appears under the "FT" key in Site objects must be described here. Each key gives the filter name and each value is an object with at least a "Desc" key giving the filter's description.

3.5 Model

Type String

Description The name, or a description, of the genotyping model that was used.

3.6 Child_Map

Type Object

Description Records parent/site relationships between variant sites and on which haplogroups child sites lie.

Each key corresponds to a site index in the Sites array. The value associated to each key is a Child listing object.

Starting from each entry of the Lvl1_Sites, the Child_Map can be recursively traversed to recover the full parent/child site structure of the graph.

3.6.1 Child listing

A Child listing is an object whose keys are haplogroups and whose values are arrays of site indices. It records, for a given parent site, what child sites it has, and which haplogroups the child sites fall under.

3.7 Lvl1_Sites

Type Array

Description Lists the indices of sites which are not nested in others.

Each element of this array corresponds to a site which has no parents; the value is the index at which to access this site in the Sites array.

Each site referred to in this array may or may not have children, depending on whether or not it appears in Child_Map.
